# Blockade of translationally controlled tumor protein attenuated the aggressiveness of fibroblast-like synoviocytes and ameliorated collagen-induced arthritis

**DOI:** 10.1038/s12276-020-00546-y

**Published:** 2021-01-06

**Authors:** Mingyo Kim, Yongho Choe, Heewon Lee, Min-Gyu Jeon, Jin-Ho Park, Hae Sook Noh, Yun-Hong Cheon, Hee Jin Park, Jaehun Park, Sung Jae Shin, Kyunglim Lee, Sang-Il Lee

**Affiliations:** 1 Department of Internal Medicine and Institute of Health Science, Gyeongsang National University School of Medicine and Hospital, Jinju, 52727 Republic of Korea; 2 Graduate School of Pharmaceutical Sciences, College of Pharmacy, Ewha Womans University, Seoul, 03760 Republic of Korea; 3 Department of Microbiology, Institute for Immunology and Immunological Disease, Brain Korea 21 PLUS Project for Medical Science, Yonsei University College of Medicine, Seoul, 03722 Republic of Korea

**Keywords:** Translational research, Autoimmunity

## Abstract

Histamine releasing factor/translationally controlled tumor protein (HRF/TCTP) stimulates cancer progression and allergic responses, but the role of HRF/TCTP in rheumatoid arthritis (RA) remains undefined. In this study, we explored the pathogenic significance of HRF/TCTP and evaluated the therapeutic effects of HRF/TCTP blockade in RA. HRF/TCTP transgenic (TG) and knockdown (KD) mice with collagen-induced arthritis (CIA) were used to determine the experimental phenotypes of RA. HRF/TCTP levels in the sera of RA patients were measured and compared to those from patients with osteoarthritis (OA), ankylosing spondylitis, Behçet’s disease, and healthy controls. HRF/TCTP expression was also assessed in the synovium and fibroblast-like synoviocytes (FLSs) obtained from RA or OA patients. Finally, we assessed the effects of HRF/TCTP and dimerized HRF/TCTP-binding peptide-2 (dTBP2), an HRF/TCTP inhibitor, in RA-FLSs and CIA mice. Our clinical, radiological, histological, and biochemical analyses indicate that inflammatory responses and joint destruction were increased in HRF/TCTP TG mice and decreased in KD mice compared to wild-type littermates. HRF/TCTP levels in the sera, synovial fluid, synovium, and FLSs were higher in patients with RA than in control groups. Serum levels of HRF/TCTP correlated well with RA disease activity. The tumor-like aggressiveness of RA-FLSs was exacerbated by HRF/TCTP stimulation and ameliorated by dTBP2 treatment. dTBP2 exerted protective and therapeutic effects in CIA mice and had no detrimental effects in a murine tuberculosis model. Our results indicate that HRF/TCTP is a novel biomarker and therapeutic target for the diagnosis and treatment of RA.

## Rheumatoid arthritis: A problem protein to target for treatment

A protein known to stimulate cancer and allergies has been implicated as an exacerbating factor in rheumatoid arthritis (RA); inhibiting its activity or blocking its production could offer options to prevent or treat RA. Previous research had identified high levels of histamine-releasing factor (HRF), also known as translationally controlled tumor protein (TCTP), in patients with RA. Its role in the destruction of joint tissue seen in RA was investigated by researchers in South Korea led by Sang-Il Lee at Gyeongsang National University, Jinju, and Kyunglim Lee at Ewha Womans University, Seoul. They examined HRF/TCTP’s activity and effects in fibroblast-like synoviocytes from RA patients and in a mouse model of RA and confirmed it can cause joint inflammation and damage. A molecule that inhibits HRF/TCTP activity reduced the severity of RA-like effects, suggesting potential new therapies.

## Introduction

Rheumatoid arthritis (RA), a chronic inflammatory and systemic autoimmune disease that affects 1% of the general population^1^, is characterized by destructive joint inflammation and systemic comorbidities, including cardiovascular complications, psychological impairment, and risk of malignancy^2–4^. Our understanding of RA pathogenesis has improved, and therapeutic strategies against RA have been revolutionized by the development of targeted drugs such as tumor necrosis factor inhibitors, interleukin-6 (IL-6) blockers, B-cell depletion agents, and inhibitors of T-cell costimulation and Janus kinases^5^. Nevertheless, an appreciable percentage of patients experience refractory disease and interrupt treatment due to the risk of infection and malignancy^6,7^. Meanwhile, autoantibodies may be present long before the onset of clinical arthritis; thus, recent investigations have focused on RA in preclinical stages^8,9^. Although several strategies are currently being tested, there are still no effective methods of RA prevention. Thus, the investigation of new cytokines or cellular mechanisms that could be potential targets for RA treatment or prevention remains of high importance.

Pannus at the interface between the synovium, cartilage, and bone is a characteristic of RA. Therefore, pannus provides a microenvironment that sustains autoimmunity and supports cellular and cytokine activities crucial for joint destruction^10,11^. Fibroblast-like synoviocytes (FLSs), which are the major components of hyperplastic pannus, resemble cancer cells in their resistance to apoptosis, increased migratory and invasive properties, and ability to escape contact inhibition. FLSs can destroy bone and cartilage even after their removal from the autoimmune milieu^12–14^. The connective-tissue origin of FLSs may render them intrinsically resistant to current immunosuppressive drugs. Thus, recurrent or refractory RA likely depends on the persistent immunopathologic activity of resident RA-FLSs within pannus^15^. Disrupting FLS-specific immunopathology may prevent joint destruction by mechanisms that do not contribute to systemic immunosuppression and increased infection risk^16–18^. Accordingly, interfering with RA-FLS proliferation and invasion may contribute to effective and long-term disease control.

Histamine releasing factor (HRF) was initially classified as a tumor protein (translationally controlled tumor protein, TCTP) in the 1980s, but its function remains unclear^19,20^. HRF/TCTP triggers histamine release from basophils; however, HRF/TCTP also functions similarly to proinflammatory cytokines in several inflammatory and allergic diseases, including asthma, atopic dermatitis, and eosinophilic pneumonia^21–23^. A previous study demonstrated that HRF/TCTP inhibits p53-mediated apoptosis, indicating that HRF/TCTP plays a distinctive role in tumorigenicity^24^. This notion is supported by our previous study, which shows that HRF/TCTP induces epithelial-mesenchymal transition (EMT) and promotes the pulmonary metastasis of melanoma cells^25^. Therefore, HRF/TCTP is a promising target for prostate, breast, and lung cancer therapies^26^. Because RA is a representative inflammatory disease and RA-FLSs exhibit p53 dysfunction and undergo a tumor-like EMT process, HRF/TCTP may potentially serve as a novel therapeutic target in the treatment of RA.

An earlier study showed that HRF/TCTP is present and localized in the joints of only patients with RA and not those of healthy controls. In particular, HRF/TCTP expression was more pronounced in the FLSs of severely destroyed regions of the pannus in RA samples^27^. This finding strongly suggests that HRF/TCTP expression contributes to RA pathogenesis; however, no other studies have examined the functions or relevance of HRF/TCTP in RA. Here, we explored the role of HRF/TCTP in RA using HRF/TCTP transgenic (TG) and knockdown (KD) mice with collagen-induced arthritis (CIA). We also assessed the levels of HRF/TCTP in the sera, joint fluid, synovial tissue, and FLSs of patients with RA and controls and examined how HRF/TCTP expression correlates with RA disease activity. Additionally, we evaluated the inhibitory effects of HRF/TCTP-binding peptide on the RA-FLS phenotype and assessed the preventive and therapeutic effects of this peptide in mice with CIA.

## Materials and methods

### Patients

Patients with RA were classified according to the ACR/EULAR 2010 Rheumatoid Arthritis Classification Criteria^28^. RA disease activity was determined with the disease activity score (DAS28) using clinical and laboratory data^29^. Patients with osteoarthritis, ankylosing spondylitis, and Behçet’s disease were classified according to specific disease classification criteria^30–32^. All patients provided written informed consent. The biospecimens used in this study were provided by Gyeongsang National University Hospital, a member of the Korea Biobank Network. This study was approved by the Institutional Review Board of Gyeongsang National University Hospital (permit No: GNUH 2014-02-013).

### Animals and experimental protocols

All mouse experimental procedures were approved by the Institutional Animal Care and Use Committee of Gyeongsang National University (permit No: GNU-150907-M0049). HRF/TCTP transgenic (TG) mice were generated as described in the “Materials and methods”. HRF/TCTP knockdown (KD) mice, heterozygous for deletion of the HRF/TCTP allele, were kindly provided by Hsin-Fang Yang-Yen (Institute of Molecular Biology, Academia Sinica, Taipei, Taiwan). The generation of HRF/TCTP KD mice is described in the “Materials and methods” section.

A previously established protocol with slight modifications was used to induce CIA using C57BL/6 strain mice^33,34^. Wild-type (WT), TG, or KD mice (male, 14–18 weeks old) were immunized with 150 μg of chicken type II collagen (CII; Chondrex, Redmond, WA, USA) emulsified in an equal volume of 4 mg/ml complete Freund’s adjuvant (CFA; Chondrex). The day of initial immunization was designated day 0. Immunization was boosted by an equal volume of emulsion of CII and incomplete Freund’s adjuvant (IFA; Chondrex) on day 21 and the intraperitoneal (i.p.) injection of lipopolysaccharide (LPS, 5 μg/mouse; Sigma-Aldrich, Saint Louis, MO, USA) on day 28. Clinical arthritis scores, ankle thickness, and micro-computed tomography (micro-CT) imaging were evaluated as described in the “Materials and methods”.

To evaluate the preventive and therapeutic effects of dimerized HRF/TCTP-binding peptide (dTBP2) in the CIA model, dTBP2 was prepared as described in the “Materials and methods”. DBA/1 mice (male, 7–9 weeks old) were immunized with 100 μg of bovine type II collagen (BII; Chondrex) emulsified with an equal volume of 2 mg/mL CFA. Immunization was boosted with an equal amount of BII emulsified in IFA on day 21. For the preventive approach, CIA mice were administered daily i.p. injections of vehicle or dTBP2 (5, 12.5, or 25 mg/kg) from day 21 until the end of the experiment. For the therapeutic approach, following the development of overt arthritis (average score of 5) on day 41, CIA mice were administered daily i.p. injections of vehicle or dTBP2 (5 or 25 mg/kg) from day 41 until the end of the experiment. Measurement of the arthritis score and ankle thickness and histopathological examination were carried out as described in the “Materials and methods”.

### Generation of HRF/TCTP transgenic (TG) and knockdown (KD) mice

HRF/TCTP TG mice were generated in the C57BL/6 and CBA hybrid background using the targeting cDNA construct pCAGGS-HRF/TCTP, which contained the CMV-IE and chicken β-actin promoter, as previously described^35^, and were then backcrossed with C57BL/6 mice. Mice heterozygous for deletion of the TCTP allele, TCTP+/−, were then backcrossed with C57BL/6 mice. HRF/TCTP KD mice heterozygous for deletion of the HRF/TCTP allele were kindly provided by Hsin-Fang Yang-Yen (Institute of Molecular Biology, Academia Sinica, Taipei, Taiwan). After the construction and electroporation of the targeting vector into R1 ES cells, the positive clones were transiently transfected with a cytomegalovirus promoter-driven Cre expression vector to obtain subclones either with the floxed HRF/TCTP allele or in which the HRF/TCTP was absent. The obtained ES cell clones were microinjected into mouse blastocysts, and the mice were backcrossed to generate HRF/TCTP KD mice.

### Arthritis scoring and ankle thickness measurement

Clinical arthritis scores on a scale of 0–4 were evaluated for each limb (0 = no swelling; 1 = slight swelling and erythema; 2 = moderate swelling and erythema; 3 = severe swelling and erythema; and 4 = maximal inflammation with joint rigidity). The maximum possible score for each mouse was 16. Hind paw thickness was measured using an electric caliper placed at the widest point across the ankle joint.

### Micro-computed tomography (micro-CT) imaging

A SkyScan 1076 micro-CT apparatus (Bruker, Kontich, Belgium) was used to evaluate structural changes in the ankle joints of CIA mice. Ankle joints were scanned and reconstructed into a 3-dimensional structure with a voxel size of 18 μm. The projections were reconstructed into 3-dimensional images using NRecon software (version 1.6.1.5) and CT Analyzer (version 1.10.0.1) (both from Bruker).

### Preparation of dimerized HRF/TCTP(d-H/T) and dimerized HRF/TCTP-binding peptide

d-H/T was prepared as described previously^36^. In brief, *Escherichia coli* BL21 (DE3) pLysS cells were transformed using pRSET A/mutated d-H/F, and the overexpressed d-H/F was then purified using a Ni2^+^-charged His-Bind column according to the manufacturer’s protocol (ELPIS, Daejeon, Korea). NH2-terminal fusion proteins of d-H/T were separated by fast protein liquid chromatography on a HiTrap Q column (GE Healthcare, Piscataway, NJ, USA) using a NaCl gradient. Synthetic dTBP2 at a purity of over 98% was provided by A & PEP Inc (Cheungju, Korea).

### Isolation and culture of fibroblast-like synoviocytes (FLSs)

Fibroblast-like synoviocytes (FLSs) were isolated from the synovial tissues of ten RA or three OA patients during total knee replacement surgery. The FLSs of OA patients were used as a control for the detection of monomeric and dimeric HRF/TCTP. The synovial tissues were minced and treated for 4 h with 2.5 mg/mL type I collagenase (Sigma-Aldrich, Saint Louis, MO, USA) in Dulbecco’s modified Eagle’s medium (DMEM) at 37 °C in 5% CO_2_. The dissociated cells were then centrifuged at 1000 × *g*, resuspended in DMEM supplemented with 10% fetal bovine serum (FBS) and 2.5 µg/ml amphotericin B, and seeded in 75 cm^2^ flasks (BD, Franklin Lakes, NJ, USA). After overnight culture, nonadherent cells were removed, and adherent cells were cultured in DMEM supplemented with 10% FBS. The cultures were maintained at 37 °C in 5% CO_2_, and the medium was replaced every 3 d. The purity of the cells was tested by flow cytometric analysis with fluorescein isothiocyanate phycoerythrin-conjugated anti-CD3, anti-CD14, anti-CD19 (all from Thermo Fisher Scientific, Waltham, MA, USA), or anti-CD90 (BD, Franklin Lakes, NJ, USA) monoclonal antibodies. A FACSCalibur flow cytometer (488Ex/620Em) was used for the analysis. Among the RA-FLSs from ten different donors, we selected those from 3, and the cells from passages 3–8 were used for each experiment.

### Cell proliferation, wound closure, and cell invasion assays

FLSs were obtained from the synovial tissues of patients with RA or OA as described in the “Materials and methods”. Dimerized HRF/TCTP (d-H/T) and dimerized HRF/TCTP-binding peptide (dTBP2) were prepared as described in the “Materials and methods“. RA-FLSs were seeded at 5 × 10^3^ cells/well in 96-well plates. After incubation for 72 h, the media were replaced with DMEM containing 0.1% FBS supplemented with 75 nM d-H/T or 75 nM d-H/T plus 75 nM dTBP2. The rate of cell proliferation was measured using the Cell Counting Kit-8 (CCK-8; Dojindo Laboratories, Kumamoto, Japan) assay according to the manufacturer’s instructions. Wound closure and cell invasion assays were performed as described previously (Jeon et al.^37^). In brief, RA-FLS monolayers grown to confluence on 60-mm culture dishes were scratched with a sterile 200-μl pipette tip and then treated with 75 nM d-H/T or 75 nM d-H/T plus 75 nM dTBP2. The width of the wounded area was imaged and measured under an inverted phase-contrast microscope (Nikon) at ×50 magnification to assess cell migration at 0 and 48 h after scratching. To examine cell invasiveness, Transwell migration assay chambers (BD, Franklin Lakes, NJ, USA) with an 8-μm pore size were used for a two-chamber migration assay. The upper surfaces of the Transwell inserts were coated with Matrigel (BD; 50 mg/filter), and RA-FLSs were seeded using serum-free medium. Complete growth medium supplemented with 75 nM d-H/T or 75 nM d-H/T plus 75 nM dTBP2 was added to the lower chamber as a chemoattractant. After 24 h, cells that had invaded and passed through the bottom surface of the insert were fixed with a 10% formalin solution, stained with propidium iodide (Invitrogen, Carlsbad, CA, USA), and quantified by counting the cells in 10 randomly selected viewing fields.

### RNA isolation and quantitative real-time (qRT)-PCR

Total RNA extracted from frozen joint tissues using TRIzol (Invitrogen, Carlsbad, CA, USA) was subjected to cDNA synthesis using the iScript cDNA synthesis kit (Bio-Rad, Hercules, CA, USA) according to the manufacturer’s instructions. qRT-PCR was performed on a ViiA 7 real-time PCR system (Applied Biosystems, Foster City, CA) using TaqMan Universal PCR Master Mix (Applied Biosystems) and TaqMan primer/probe sets (Pre-Developed Assay Reagents; Applied Biosystems) for IL-1β, IL-6, MMP-1, MMP-3, MMP13, and CCR2 (all as instructed by the manufacturer). Data were normalized to the expression of *GAPDH* (an internal control), and relative expression was calculated using the 2^−ΔΔCt^ method.

### Histopathological examination

Ankle joint tissues were harvested, fixed in 10% formalin, decalcified for 4 weeks in 10% EDTA, embedded in paraffin, and sectioned into 5 μm sections. The sections were then stained with hematoxylin and eosin (H&E), safranin O, or tartrate-resistant acid phosphatase (TRAP). Synovial inflammation, cartilage damage, and bone erosion were evaluated, and TRAP-positive multinucleated osteoclasts (with ≥3 nuclei) were counted as described previously^37^.

### Immunohistochemical and immunofluorescent staining

For immunohistochemical staining, human synovial tissue sections were incubated with an anti-HRF/TCTP antibody (1:100; Abcam, Cambridge, UK) overnight at 4 °C. Sections were then incubated with biotinylated anti-rabbit IgG (Vector Laboratories, Burlingame, CA, USA) for 1 h at room temperature. HRF/TCTP-positive cells were detected using a Peroxidase-Conjugated Avidin-Biotin Complex kit (Vector Laboratories), followed by staining with 3′3-diaminobenzidine tetrahydrochloride (DAB; Sigma-Aldrich, Saint Louis, MO, USA). For immunofluorescent staining, tissue sections were incubated with monoclonal anti-CD55 antibody (1:100; Santa Cruz Biotechnology, Dallas, TX, USA) plus anti-HRF/TCTP antibody (1:100; Abcam) overnight at 4 °C. Each slide was then incubated with Alexa Fluor 594-conjugated anti-rabbit IgG or Alexa 488-conjugated anti-mouse IgG (both from Thermo Fisher Scientific, Waltham, MA, USA). Fluorescence images were obtained using a fluorescence microscope (BX51, Olympus) at excitation/emission wavelengths of 488/520 nm (for Alexa Fluor 488) or 594/617 nm (for Alexa Fluor 594).

### Enzyme-linked immunosorbent assay (ELISA)

The levels of HRF/TCTP (LSBio, Seattle, WA, USA), anti-collagen IgG, IgG1, and IgG2a (R&D Systems, Minneapolis, MN, USA) in the sera, synovial fluid, and FLS culture supernatants were measured using ELISA kits according to the instructions of the corresponding manufacturer.

### Western blot analysis

Total proteins were extracted from FLSs with RIPA lysis buffer supplemented with protease inhibitor cocktail (Calbiochem, San Diego, CA, USA). Nuclear fractionation was performed using NE-PER Nuclear and Cytoplasmic Extraction Reagents (Thermo Fisher Scientific, Waltham, MA, USA). Total protein lysates or nuclear extracts were separated by SDS-PAGE and transferred onto a membrane. For dimerized HRF/TCTP detection, proteins were fractionated by nonreducing SDS-PAGE. The membranes were blocked with 5% skimmed milk and incubated with primary antibodies against the following: HRF/TCTP (Abcam, Cambridge, UK), β-actin (GenDEPOT, Katy, TX, USA), ERK, phospho-ERK, phospho-IκBα, Lamin A/C (all Cell Signaling, Danvers, MA, USA), GAPDH (ABFrontier, Seoul, Korea), and p65 (Enzo Life Sciences, Farmingdale, NY, USA). Signals were visualized using the corresponding HRP-conjugated secondary antibodies and substrates. Bands were normalized using a loading control and quantified by densitometry using ImageJ 1.48 software.

### *Mycobacterium tuberculosis* (Mtb) culture

Mtb H37Rv (ATCC 27294) was purchased from American Type Culture Collection (ATCC, Manassas, VA, USA). The original stock was streaked on a plate containing 7H11 medium to which OADC had been added and incubated at 37 °C. After 3–4 weeks, a single colony was picked and incubated for 2 weeks in a culture flask containing 7H9 broth supplemented with OADC. To this culture flask, 7H9 broth containing OADC was added, after which the flask was incubated at 37 °C for 7‒10 days. The flask was incubated for another 7‒10 days, after which single cells were prepared. The seed lots for each strain were stored in small aliquots at −80 °C until use.

### Mtb infection and treatment

Specific pathogen-free 6-week-old C57BL/6J female mice were purchased from Japan SLC, Inc. (Shijuoka, Japan) and strictly maintained under barrier conditions in a Biosafety Level 3 (BSL-3) facility at the Avison Biomedical Research Center at the Yonsei University College of Medicine (Seoul, Korea). All animal studies were performed in accordance with Korean Food and Drug Administration (KFDA) guidelines. The experimental protocols used in this study were approved by the Ethics Committee and Institutional Animal Care and Use Committee of the Laboratory Animal Research Center at Yonsei University College of Medicine (permit number: 2017-0342).

Mice were infected with Mtb strain H37Rv via aerosol^38^. The mice were exposed to the Mtb strain H37Rv for 60 min in the calibrated inhalation chamber of an airborne infection apparatus that delivers a predetermined Mtb dose (Glas-Col, Terre Haute, IN, USA), and ~150 viable bacteria were delivered into the lungs. To confirm the initial bacterial burden, the mice were euthanized one day later, and ~150 viable bacteria were delivered to the lungs of each mouse. Mouse anti-TNF-α monoclonal antibody (MP6-XT22; BioLegend, San Diego, CA) in PBS was administered intraperitoneally (0.25 mg/mouse; 200 µl/mouse) for neutralization once before infection and once every week for 4 weeks after Mtb infection. dTBP2 (50 mg/kg) in PBS was administered intraperitoneally (200 µl/mouse) twice per week for 4 weeks after infection. All mice were euthanized 30 d after Mtb infection.

### Histopathological analysis and enumeration of Mtb

Disease phenotype and severity were evaluated through histopathology and by measuring bacterial growth in the lung and spleen. To determine how well they had been protected, the organs were removed 30 d after infection. For lung histopathology, the right superior lobes were fixed overnight in 10% formalin and embedded in paraffin. Lungs were sectioned at a thickness of 4–5 μm and stained with H&E. For bacterial growth analysis, the lung and spleen were homogenized, and serially diluted samples were plated onto Middlebrook 7H10 agar plates (Becton Dickinson, Franklin Lakes, NJ) supplemented with 10% OADC (Difco Laboratories, Detroit, VA), 2 μg/ml 2-thiophenecarboxylic acid hydrazide (Sigma-Aldrich, St. Louis, MO) and amphotericin B (Sigma-Aldrich). After 4 weeks of incubation at 37 °C, bacterial colonies were counted. Data are presented as log_10_ colony-forming units (CFU) per organ.

### Statistical analyses

Data were analyzed using two-way analysis of variance (ANOVA) followed by Fisher’s post hoc analysis. The significance of differences between groups was determined using Student’s unpaired *t*-test or one-way ANOVA with Tukey’s post hoc test. Data are expressed as the means ± SEMs, and a *p*-value of less than 0.05 was used to indicate statistical significance.

## Results

### HRF/TCTP overexpression and knockdown were correlated with arthritic phenotypes in mice with CIA

**Fig. 1:**
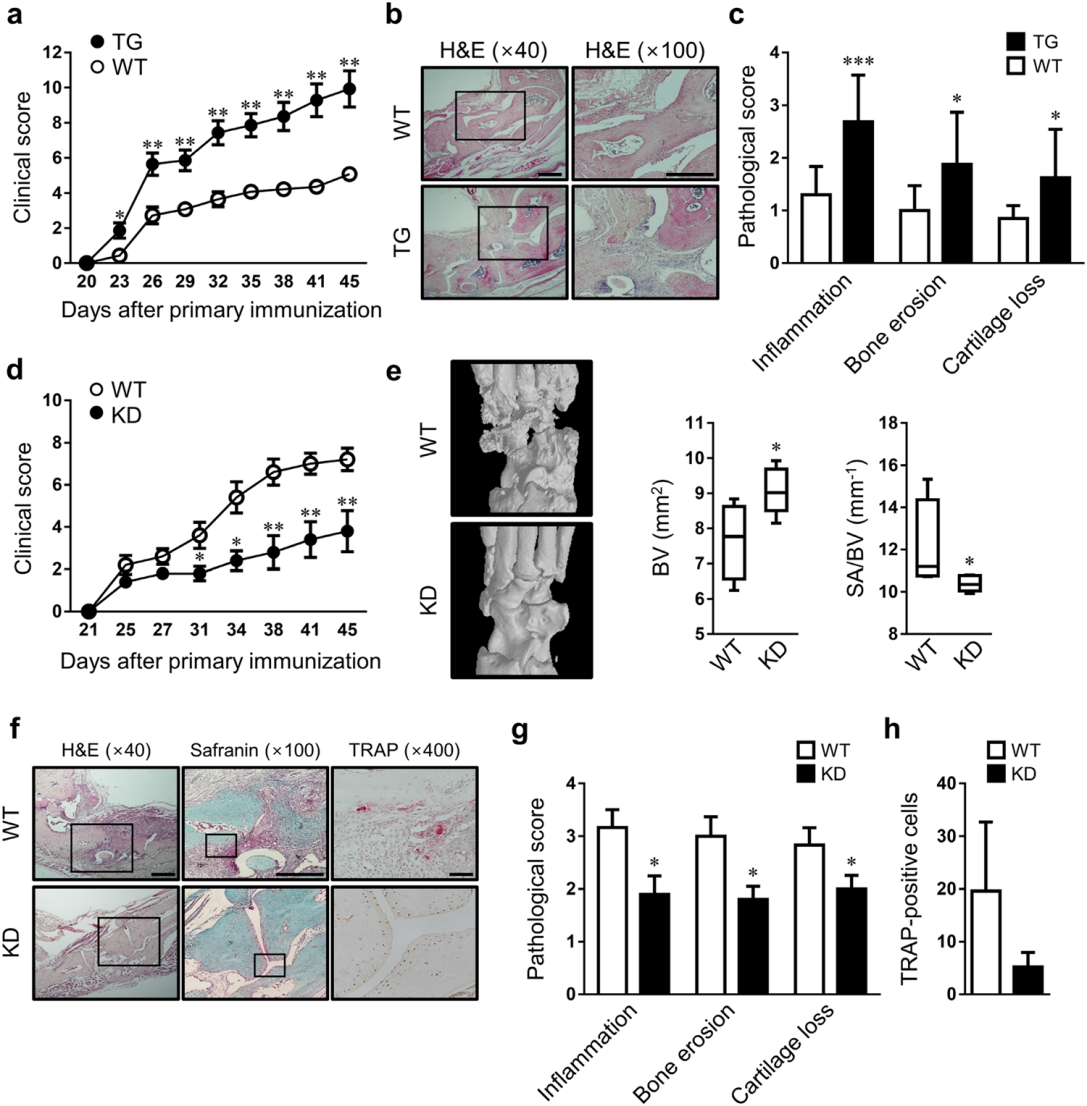
Role of HRF/TCTP in autoimmune arthritis. **a** Mean clinical scores of HRF/TCTP transgenic (TG) mice and wild-type (WT) littermates after the generation of collagen-induced arthritis (CIA) (*n* = 7 mice for each group). **b** Representative images of ankle joint tissue harvested from TG and WT mice and stained with hematoxylin and eosin (H&E), showing changes in tissue morphology typical of CIA (bar = 500 μm). **c** Pathological scores for synovial inflammation, cartilage damage, and bone erosion for the ankle joints of CIA mice (*n* = 14 ankles for each group). **d** Mean clinical scores of HRF/TCTP-knockdown (KD) and WT mice after CIA induction (*n* = 5 mice for each group). **e** Micro-CT images of hind paws (left), the percentage of bone volume (BV), and the ratio of the bone surface to bone volume (BS/BV) (right). **f** Representative images of ankle joint tissue harvested from KD and WT mice and stained with H&E and safranin O (bar = 500 μm) or immunolabeled for TRAP (bar = 50 μm)**. g**, **h** Pathological scores (**g**) and the number of TRAP-positive cells (**h**) (*n* = 10 ankles for each group). A high-magnification view of the boxed area is presented in the right panel. Values are the mean ± SEM; *p < 0.05; **p < 0.01; ***p < 0.001 versus WT.

We established a CIA model in transgenic (TG) mice to examine the role of HRF/TCTP overexpression in RA. CIA was significantly more severe in TG mice than in WT littermates (Fig. 1a). Assessment of joint pathology revealed that joint inflammation, bone erosion, and cartilage destruction in the TG mice were more pronounced than those in the WT mice (Fig. 1b, c). We then examined whether HRF/TCTP knockdown (KD) would exert an antiarthritic effect upon CIA generation. As expected, KD mice showed an attenuated arthritic phenotype with respect to the WT mice (Fig. 1d). Micro-CT and pathological data demonstrated reduced bone destruction in KD mice compared with WT mice (Fig. 1e). Similarly, synovial inflammation, bone erosion, cartilage damage, and osteoclastic bone resorption were less pronounced in KD mice than in WT mice (Fig. 1f–h). These results show that HRF/TCTP enhanced the manifestation of CIA, but knockdown of HRF/TCTP inhibited the arthritic inflammation and joint destruction observed in CIA.

### HRF/TCTP was highly expressed and positively correlated with disease activity in patients with RA

**Fig. 2:**
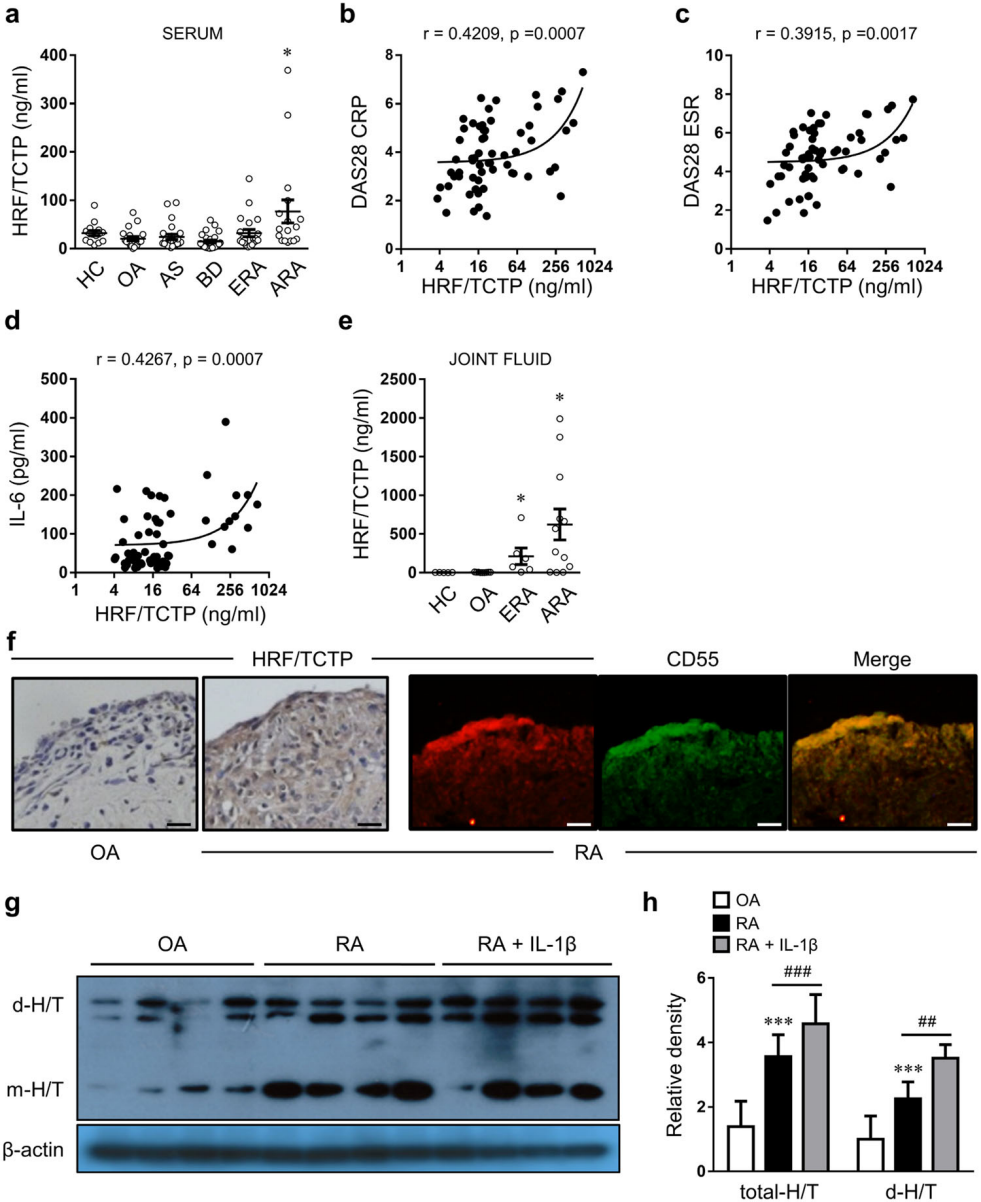
Levels of HRF/TCTP in patients with rheumatoid arthritis (RA) and their relationship with disease severity. **a** Levels of HRF/TCTP in the sera of healthy controls (HC, *n* = 15) and patients with osteoarthritis (OA, *n* = 20), ankylosing spondylitis (AS, *n* = 23), Behçet’s disease (BD, *n* = 26), early RA (ERA, disease duration <1 year, *n* = 21), and advanced RA (ARA, disease duration ≥1 year, *n* = 17), as determined by ELISA. **b**–**d** Correlation of serum HRF/TCTP levels with DAS28-CRP (*n* = 62) (**b**), DAS28-ESR (*n* = 62) (**c**), and serum IL-6 concentration in patients with RA (*n* = 60) (**d**). **e** Levels of HRF/TCTP in the joint fluids of healthy controls (HC, *n* = 5) and patients with osteoarthritis (OA, *n* = 9), early RA (ERA, *n* = 6), and advanced RA (ARA, *n* = 12). **f** HRF/TCTP expression in synovial tissue from patients with OA and RA, as determined by immunohistochemistry (left) and double immunofluorescence labeling (right) for CD55 (green) and HRF/TCTP (red). Scale bar: 100 μm. **g**, **h** Protein expression of the monomeric and dimeric forms of HRF/TCTP in OA-FLSs, RA-FLSs, and IL-1β-stimulated RA-FLSs, as determined by western blotting (**g**); relative density normalized to that of β-actin (**h**). Values are the mean ± SEM; *p < 0.05; ***p < 0.001 versus healthy controls (**a**, **e**) or OA-FLSs (**h**); ^##^*p* < 0.01; ^###^*p* < 0.001 versus the value before IL-1β stimulation (**h**).

To further determine the role of HRF/TCTP in human RA, we next assessed HRF/TCTP levels in human blood and joint fluid. While HRF/TCTP levels in other inflammatory diseases were similar to those observed in healthy individuals and patients with osteoarthritis (OA), only patients with advanced RA exhibited high serum levels of HRF/TCTP in the present study (Fig. 2a). Additionally, HRF/TCTP levels were positively correlated with DAS28-CRP, DAS28-ESR, and the serum levels of IL-6 in patients with RA (Fig. 2b–d). Furthermore, HRF/TCTP levels in joint fluid were higher in patients with the early stages of RA and significantly higher in patients with advanced RA than in patients with OA and healthy individuals employed as controls (Fig. 2e). Because the concentration of HRF/TCTP in the joint fluid was 10-fold greater than that in the serum, we hypothesized that the cells mainly responsible for secreting HRF/TCTP reside in the joint. Thus, we examined HRF/TCTP expression in human joint tissues. We found that the expression of HRF/TCTP was higher in the RA synovium than in the OA synovium. This finding is supported by the finding that CD55-positive RA-FLSs, which represent the predominant cell type in RA synovium, were costained for HRF/TCTP (Fig. 2f). The expression of HRF/TCTP, particularly the active dimer form of HRF/TCTP (d-H/T), was higher in RA-FLSs than in OA-FLSs and further increased by stimulation with IL-1β (Fig. 2g, h). Taken together, these results indicate that RA-FLSs stimulated by the inflammatory environment can robustly secrete HRF/TCTP. This result explains why the levels of HRF/TCTP were higher in patients with RA and correlated well with clinical disease activity.

### HRF/TCTP activated promigratory and invasive phenotypes in RA-FLSs

**Fig. 3:**
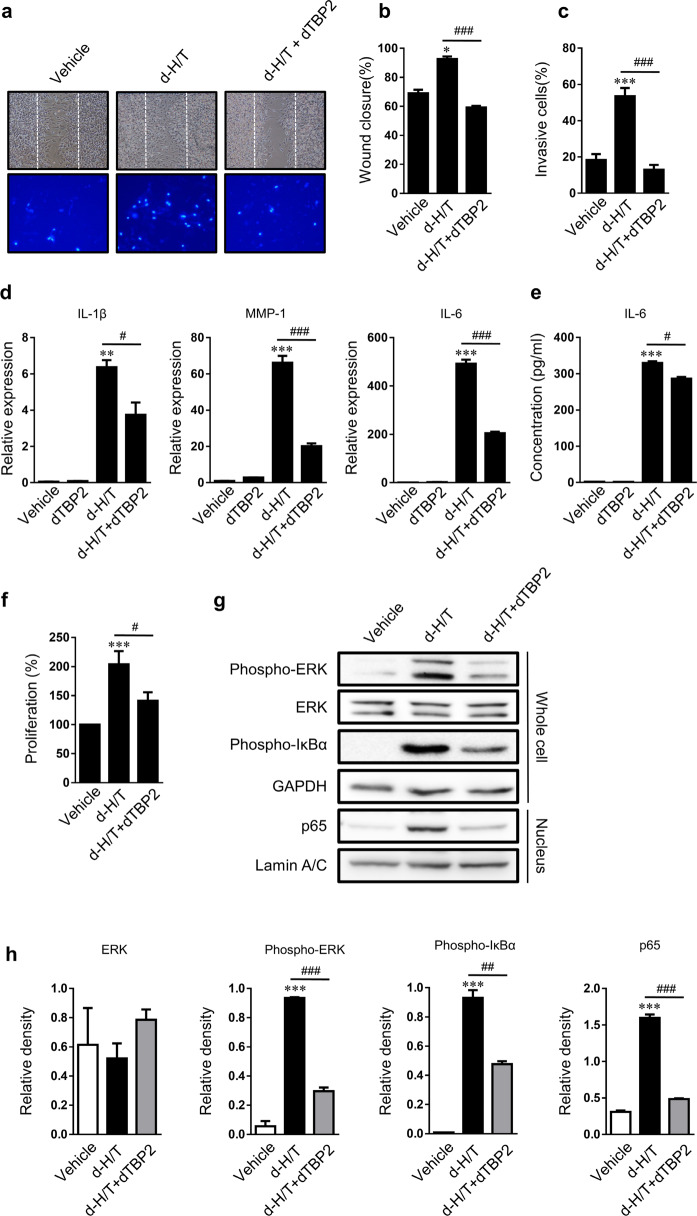
Effects of dimerized HRF/TCTP (d-H/T) and inhibitory effects of dimerized HRF/TCTP-binding peptide (dTBP2) on rheumatoid arthritis fibroblast-like synoviocytes (RA-FLSs). RA-FLSs were treated with vehicle, 75 nM d-H/T, or 75 nM d-H/T plus 75 nM dTBP2 as described in the “Materials and methods”. **a** Representative microphotographs showing cells that had migrated into the wounded area after 48 h (upper panel, ×50 magnification) and invasive cells that passed through the Matrigel-coated Transwell membrane after 24 h (lower panel, ×100 magnification). **b**, **c** Migrated (**b**) and invading (**c**) cells were quantified by counting the cells in randomly selected fields. **d**, **e** mRNA levels of IL-1β, IL-6, and MMP1 in RA-FLSs and protein levels of IL-6 in culture supernatants were determined at 12 hr by qRT-PCR (**d**) and at 24 h by ELISA (**e**) after treatment with vehicle, dTBP2, d-H/T, or d-H/T plus dTBP2. **f** Change in the proliferation of RA-FLSs treated with vehicle, d-H/T, or d-H/T with dTBP2, as measured by WST-1 assay. **g**, **h** Protein levels of ERK, phospho-ERK, and IkB in whole-cell extracts and levels of p65 in nuclear fractions, as determined by western blotting (**g**); relative density normalized to that of GAPDH or lamin A/C (**h**). Values are the mean ± SEM of three experiments; *p < 0.05; **p < 0.01; ***p < 0.001 versus vehicle-treated RA-FLSs; # p < 0.05; ### p < 0.001 versus d-H/T-treated RA-FLSs.

Next, we examined the effects of HRF/TCTP on the cancer-like phenotype of RA-FLSs. Our results show that d-H/T, an active dimerized form of HRF/TCTP, stimulated the migration, invasion, and proliferation of RA-FLSs. The blockade of d-H/T via dTBP2, an HRF/TCTP-binding peptide, decreased the aggressiveness of d-H/T-stimulated RA-FLSs (Fig. 3a–c, f). Treatment with d-H/T enhanced expression of the inflammatory cytokines IL-1β, IL-6, and MMP1, while treatment with dTBP2 abolished the increased expression of these cytokines (Fig. 3d, e). We evaluated the effect of HRF/TCTP on pathways involving RA-FLSs: d-H/T-induced increases in ERK phosphorylation in the mitogen-activated protein kinase (MAPK) pathway, the phosphorylation of IκBα, and the nuclear translocation of p65 in the NFκB pathway. As expected, these effects were blocked by dTBP2 (Fig. 3g, h). Collectively, these results show that HRF/TCTP plays a crucial role in stimulating the cancer-like aggressiveness of RA-FLSs via the ERK and p65 pathways.

### HRF/TCTP blockade prevented arthritic development in mice with CIA

**Fig. 4:**
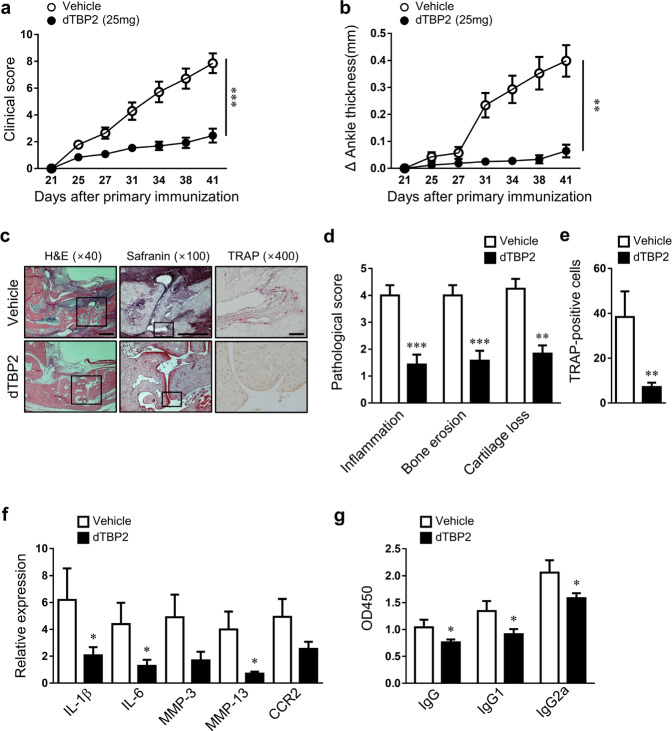
Prevention of inflammatory arthritis by dimerized HRF/TCTP-binding peptide (dTBP2). After the generation of collagen-induced arthritis (CIA), mice were treated with vehicle or dTBP2 by intraperitoneal injection starting on day 21 (the day of booster immunization) and once every day until the day of sacrifice (day 41). **a**, **b** Changes in mean clinical scores (**a**) and ankle thickness (**b**) in CIA mice treated with vehicle or 25 mg/kg dTBP2. **c** Ankle tissues from groups treated with vehicle or 25 mg/kg dTBP2 were stained using H&E and safranin O (bar = 500 μm) and immunolabeled for TRAP (bar = 50 μm). **d**, **e** Pathological scores for synovial inflammation, cartilage damage, and bone erosion (**d**) and TRAP-positive cells in the ankle joints (**e)**. **f** Relative expression of the inflammatory cytokines IL-1β, IL-6, MMP3, MMP13, and CCR2 in ankle joints, as determined by qRT-PCR. **g** Levels of anti-CII IgG, IgG1, and IgG2a in the sera of mice with CIA, as quantified by ELISA. Values are the mean ± SEM (*n* = 10–12 mice for each group); *p < 0.05; **p < 0.01; ***p < 0.001 versus vehicle-treated CIA mice.

Based on our in vitro data showing that dTBP2 dramatically inhibited the inflammatory and cancer-like characteristics of RA-FLSs, we next evaluated whether dTBP2 could prevent the development of autoimmune arthritis in a CIA mouse model. Mice with CIA treated with 12.5 or 25 mg/kg dTBP2 showed a significantly decreased clinical score and ankle thickness compared to those of CIA mice injected with the vehicle (Fig. 4a, b, Supplementary Fig. 1a, b). Histological analyses of ankle joints harvested from mice treated with 12.5 or 25 mg/kg dTBP2 showed milder arthritic characteristics (decreased joint inflammation, bone erosion, cartilage destruction, and osteoclastic bone resorption) than those of mice treated with vehicle or 5 mg/kg dTBP2 (Fig. 4c–e, Supplementary Fig. 1c–e). The expression levels of IL-1β, IL-6, MMP-3, MMP-13, and CCR2 were significantly lower in CIA mice treated with 25 mg/kg dTBP2 than in those treated with vehicle or 5 or 12.5 mg/kg dTBP2 (Fig. 4f, Supplementary Fig. 1f). The Anti-CII IgG, IgG1, and IgG2a concentrations in the sera of CIA mice treated with 25 mg/kg dTBP2 were lower than those in vehicle-injected CIA mice (Fig. 4g). These results show that dTBP2 prevented the development of joint inflammation and destruction in CIA.

### HRF/TCTP blockade exerted therapeutic effects in mice with CIA

**Fig. 5:**
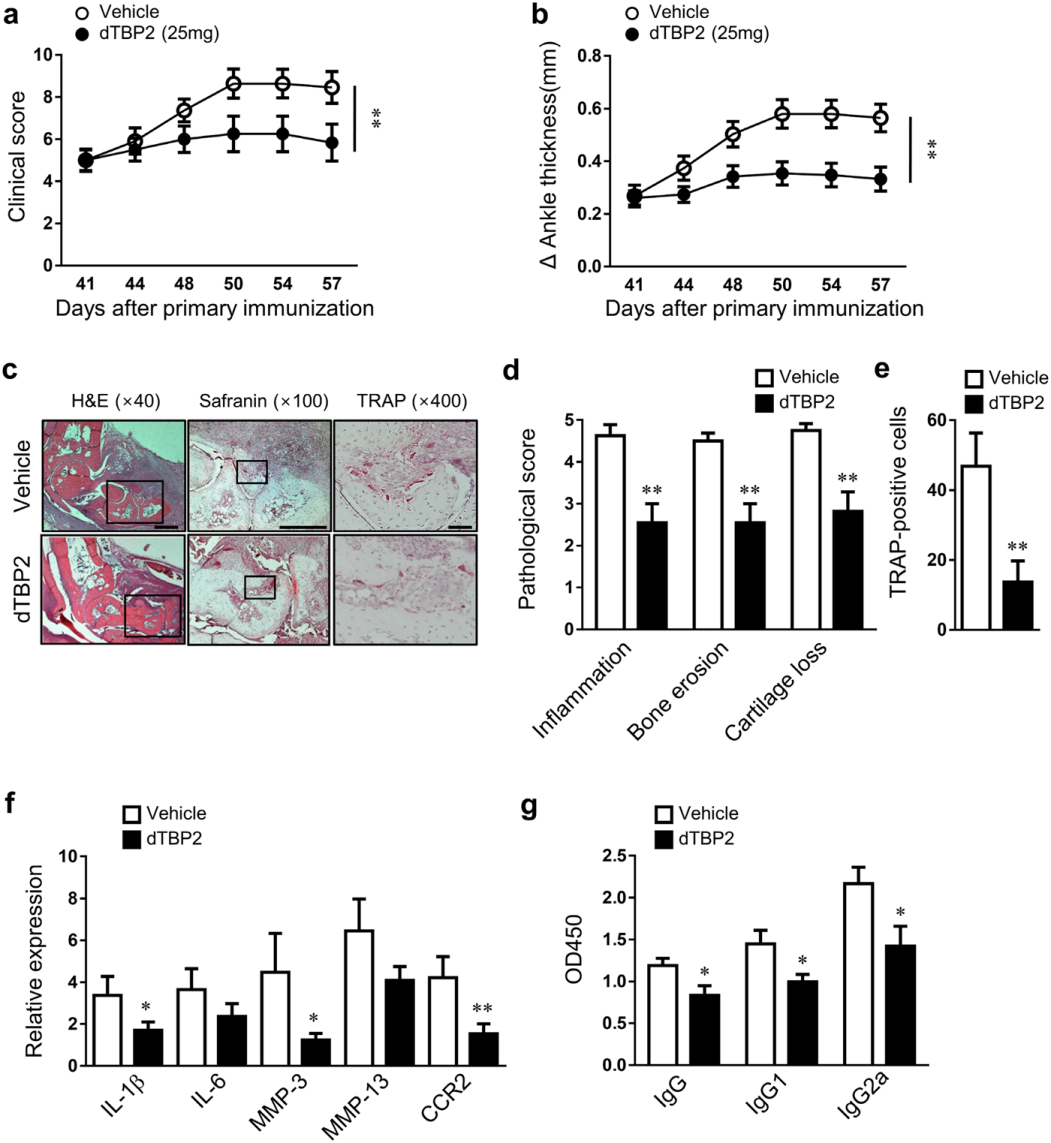
Therapeutic effects of dimerized HRF/TCTP-binding peptide (dTBP2) on inflammatory arthritis. After the generation of collagen-induced arthritis (CIA), the mice were treated with vehicle or dTBP2 by intraperitoneal injection starting on day 41 (the day at which the average arthritis score was 5) and once every day until the day of sacrifice (day 57). **a**, **b** Changes in mean clinical score (**a**) and ankle thickness **(b**) in CIA mice treated with vehicle or 25 mg/kg dTBP2. **c** Ankle tissues from groups treated with vehicle or 25 mg/kg dTBP2 were stained using H&E and safranin O (bar = 500 μm) and immunolabeled for TRAP (bar = 50 μm). **d**, **e** Pathological scores for synovial inflammation, cartilage damage, and bone erosion and TRAP-positive cells (**e**). **f, g** Relative expression of inflammatory cytokines in the ankle joint (**f**) and levels of anti-CII IgG, IgG1, and IgG2a in sera (**g**) of vehicle- or dTBP2-treated CIA mice. Values are the mean ± SEM (*n* = 10–12 mice for each group); *p < 0.05; **p < 0.01 versus vehicle-treated CIA mice.

**Fig. 6:**
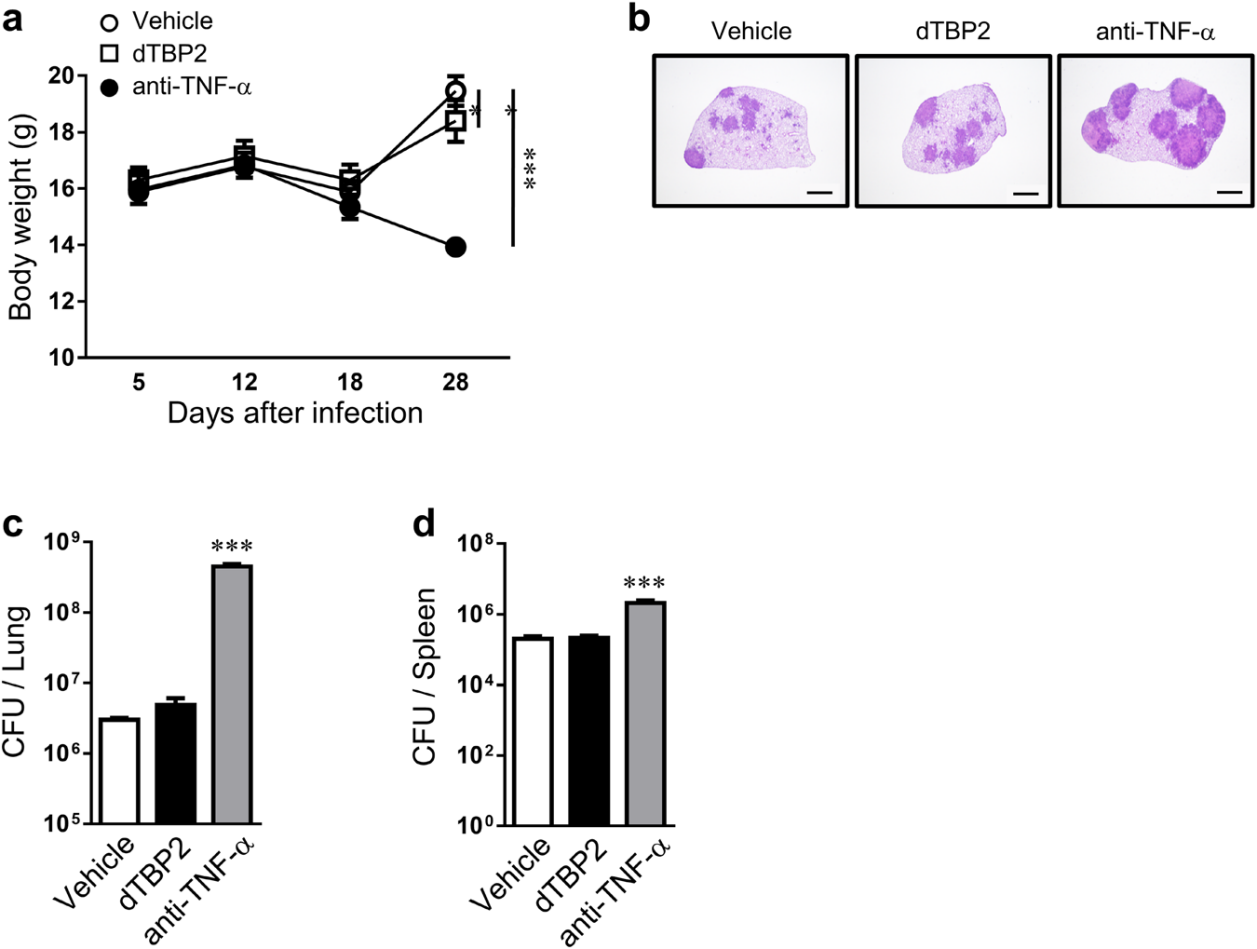
dTBP2 did not reduce host protection against Mtb infection. Mice were infected with Mtb strain H37Rv via aerosolization with 150 CFU, and the lungs were dissected 30 d postinfection for analysis. **a** Changes in body weight were measured for up to 26 d after infection. **b** The superior lobe of the right lung was stained with H&E 30 d after Mtb challenge. **c**, **d** The bacterial load in the lungs (**c**) and spleens (**d)** of mice in each group at 30 d postinfection was analyzed by counting the bacteria (bar = 2 mm). Values are the mean ± SD (*n* = 5–6 mice for each group); **p < 0.01; ***p < 0.001 versus the vehicle-treated group.

Next, we assessed the possible therapeutic effects of dTBP2 on the progression of arthritis. CIA mice (average arthritis score = 5) were injected with the vehicle or 5 or 25 mg/kg dTBP2. Mice treated with 5 mg/kg dTBP2 showed partially improved clinical scores until 54 weeks. In particular, CIA mice treated with 25 mg/kg dTBP2 showed a greater reduction in arthritic phenotypes than mice treated with the vehicle or 5 mg/kg dTBP2 (Fig. 5a, Supplementary Fig. 2a). Mice treated with 25 mg/kg dTBP2 showed a sustained decrease in ankle thickness (Fig. 5b, Supplementary Fig. 2b). Assessment of ankle joint pathology revealed that joint inflammation, bone erosion, cartilage destruction, and osteoclastic bone resorption were more attenuated in mice treated with 25 mg/kg dTBP2 than in those treated with vehicle or 5 mg/kg dTBP2 (Fig. 5c–e, Supplementary Fig. 2c–e). Compared with those of vehicle- or 5 mg/kg-treated mice, the joint tissues of mice treated with 25 mg/kg dTBP2 exhibited significantly decreased levels of IL-1β, IL-6, MMP-3, MMP-13, and CCR2 (Fig. 5f, Supplementary Fig. 2f**)**. Consistent with these results, dTBP2 treatment led to a decrease in the serum levels of anti-CII IgG, IgG1, and IgG2a (Fig. 5g). Furthermore, the reactivation of latent *Mycobacterium tuberculosis* (Mtb) infections is an important factor in the safety of RA therapeutics. In Mtb infection and treatment experiments aimed at ruling out these clinical concerns, dTBP2 decreased the severity of TB infection more than anti-TNF–α monoclonal antibody, which is used in RA treatment, and showed a phenotype similar to that of the vehicle-treated groups (Fig. 6a–d). Collectively, dTBP2 exerted preventive and therapeutic effects by downregulating the synthesis of numerous proinflammatory cytokines and antibodies, thereby slowing the progression of CIA without increasing the risk of Mtb reactivation.

## Discussion

In agreement with previous findings indicating that HRF/TCTP contributes to allergic inflammation and tumorigenesis^26,39^, in the present study, we have shown the novel role and clinical implication of HRF/TCTP expression in the pathogenesis of RA. Of multiple inflammatory diseases, HRF/TCTP is highly expressed in only RA, and its serum levels are correlated with RA disease activity. This result suggests that HRF/TCTP levels can be used as a biomarker to diagnose RA and predict its severity. In RA, HRF/TCTP is mainly produced by RA-FLSs and plays a pivotal role in conferring RA-FLSs with tumor-like capabilities, including increased migratory capacity, invasiveness, and proliferation. Experimental arthritis was promoted in HRF/TCTP TG mice and suppressed in KD mice. Inhibition of HRF/TCTP activity via dTBP2 shows preventive and therapeutic potential because it downregulates the synthesis of numerous proinflammatory cytokines and antibodies in CIA.

HRF/TCTP, as its name suggests, participates in histamine release and is mainly responsible for allergic diseases^21–23^. Interestingly, histamine is present in both the synovial tissue and joint fluid of patients with RA and recognized as a mediator of joint inflammation^40–42^. Other studies, however, indicated that histamine levels were lower in the blood and joint fluid of patients with RA than in those of controls. Additionally, prolonged treatment with anti-TNF-α significantly increased the levels of circulating histamine, suggesting that histamine is not useful as a biomarker to assess RA^43^. Conversely, a study using preclinical models of arthritis reported that both histamine H4 receptor (H4R)-deficient mice and mice treated with an H4R antagonist showed reduced arthritic severity^44^. However, a clinical trial examining toreforant, a selective H4R antagonist, was terminated prematurely due to patient fatality and the lack of significant RA alleviation^45,46^. In contrast, HRF/TCTP exhibits cytokine-like upstream functions; while it can thus potentiate histamine release, it also induces IL-8 and GM-CSF secretion, augments B-cell proliferation, and increases immunoglobulin^22^. This upstream stimulatory activity supports the correlation of HRF/TCTP expression with the clinical and inflammatory RA activities observed in our study. The targeting of HRF/TCTP may also aid in guiding future successful therapeutic approaches for RA that are similar to the use of Syk and JAK inhibitors, which target upstream signaling cascade kinase inhibitors^47^.

In humans, HRF/TCTP is generated by peripheral blood mononuclear cells, in addition to other cell types, such as T and B lymphocytes, platelets, vascular endothelial cells, and alveolar macrophages residing in tissue compartments^48^. Elevated levels of HRF/TCTP are found in the sera, bronchoalveolar lavage (BAL), and skin blister fluids of patients with asthma and various allergies^21,22^. In our study, increased levels of HRF/TCTP were found in the sera and joint fluid of patients with RA. HRF/TCTP levels were approximately 10-fold higher in the joint fluid than in the sera, which was unexpected. This finding suggests that joint stromal cells are the main source of HRF/TCTP in RA. Once activated, RA-FLSs acquire robust proliferative capacity, demonstrate tumor-like behavior, and become the most abundant stromal cell type in the inflamed joint synovium. Consistent with a previous report showing the presence of HRF/TCTP in the synovial pannus and FLSs of patients with RA^27^, our study also showed that HRF/TCTP expression was higher in the synovium and FLSs of patients with RA than in those of patients with OA. Furthermore, our results showed that IL-1β stimulation increased the production of HRF/TCTP by RA-FLSs. In agreement with previous reports, our results demonstrate that HRF/TCTP is generated mainly by RA-FLSs.

Our previous studies, as well as those of others, have recently demonstrated the presence of a dimerized form of HRF/TCTP in the sera and BAL fluids of patients and mice with atopy and asthma; additionally, dimerization appears to be essential for the proinflammatory activity of HRF/TCTP at sites of inflammation^36^. The results of our present study showed that after stimulation with IL-1β, the levels of dimerized HRF/TCTP were increased, and this active form drove the transformation of RA-FLSs into an aggressive inflammatory phenotype. MAPK and nuclear factor κB (NFκB) are involved in regulating the aggressiveness of RA-FLSs^12^. Our previous study demonstrated that dimerized HRF/TCTP induced the increased expression of IL-8, an inflammatory cytokine, via MAPK and NF-κB signaling in human bronchial epithelial cells^36^. In our present study, dimerized HRF/TCTP increased the phosphorylation of ERK and IκBα and nuclear translocation of p65 in the NFκB pathway. Thus, consistent with previous studies highlighting the importance of HRF/TCTP activity in cancer cells^26^, our results indicate a link between HRF/TCTP and the cancer-like behavior of RA-FLSs, which crucially contributes to the development and progression of RA.

Because dimerized HRF/TCTP plays such a critical role in inflammatory diseases such as RA, blocking dimerized HRF/TCTP may offer a rational strategy for the treatment of RA. For this reason, we previously searched for dimerized HRF/TCTP-binding peptides (dTBPs) by screening a phage-displayed 7-mer peptide library. One peptide, designated dTBP2, showed a stronger affinity for dimerized HRF/TCTP than for the inactive monomeric form, and dTBP2 alleviated several allergy-related symptoms in animal models of rhinitis^39^. In our present study, dTBP2 markedly inhibited the aggressiveness of RA-FLSs exacerbated by dimerized HRF/TCTP. Via both preventive and therapeutic approaches, dTBP2 robustly blocked the development and progression of arthritis in mice with CIA. The CIA model offers an opportunity to evaluate preventive treatments in RA at preclinical stages, since the prevention of RA is rather difficult to study in humans^49^. In assessing the effectiveness of new drug candidates in animal RA models, a therapeutic, rather than preventive, approach is more predictive of a drug’s clinical efficacy in human patients with RA^50,51^. In our present study, dTBP2 exerted protective and therapeutic effects in CIA mice. Thus, our results strongly suggest that the use of dTBP2 is an attractive new strategy for the prevention and treatment of RA.

In summary, our study shows that HRF/TCTP levels were increased in samples obtained from patients with RA and correlated well with RA disease activity. HRF/TCTP was found to be generated mainly by RA-FLSs, and the active dimerize form of HRF/TCTP drives RA-FLSs toward a cancer-like phenotype. Consistent with these in vitro findings, HRF/TCTP overexpression led to the exacerbation of arthritis, while HRF/TCTP knockdown attenuated arthritic manifestations in mice with CIA. dTBP2, a peptide that blocks HRF/TCTP, abolished the pathologic effects of RA-FLSs and dramatically inhibited the development and progression of arthritis in mice with CIA. Additionally, dTBP2 did not aggravate TB, while treatment with anti-TNF-α mAb aggravated TB by causing a significant increase in bacterial burden along with excessive lung inflammation in a murine TB model. Together, our findings suggest that HRF/TCTP is an attractive target and that dTBP2 has preventive and therapeutic applications in patients with RA.

## Supplementary information

Supplemental information
