# Supplementary material for: Blockade of translationally controlled tumor protein attenuated the aggressiveness of fibroblast-like synoviocytes and ameliorated collagen-induced arthritis
Source: Exp Mol Med. 2021 Jan 6;53(1):67–80. doi: 10.1038/s12276-020-00546-y (PMC8080778; doi:10.1038/s12276-020-00546-y)
Supplement: Supplementary file 1 — Supplemental information [file 12276_2020_546_MOESM1_ESM.docx]

**Blocking translationally controlled tumor protein attenuate aggressiveness of fibroblast-like synoviocytes and ameliorates collagen-induced arthritis**

**Running title**: HRF/TCTP in rheumatoid arthritis

Mingyo Kim^1^, Yongho Choe^1^, Heewon Lee,^2^, Min-Gyu Jeon^1^, Jin-Ho Park^1^, Hae Sook Noh^1^, Yun-Hong Cheon^1^, Hee Jin Park^1^, Jaehun Park^3^, Sung Jae Shin^3^, Kyunglim Lee^2,^**^¶^**

and Sang-Il Lee^1,^**^¶^**

^1^Department of Internal Medicine and Institute of Health Science, Gyeongsang National University School of Medicine and Hospital, Jinju, 52727, Republic of Korea, ^2^Graduate School of Pharmaceutical Sciences, College of Pharmacy, Ewha Womans University, Seoul, 03760, Republic of Korea, ^3^Department of Microbiology, Institute for Immunology and Immunological Disease, Brain Korea 21 PLUS Project for Medical Science, Yonsei University College of Medicine, Seoul 03722, Republic of Korea

**Contents**

1. Supplementary Figure
2. Supplementary Figure legend

**1. Supplementary figure**

**Supplementary figure 1**

**
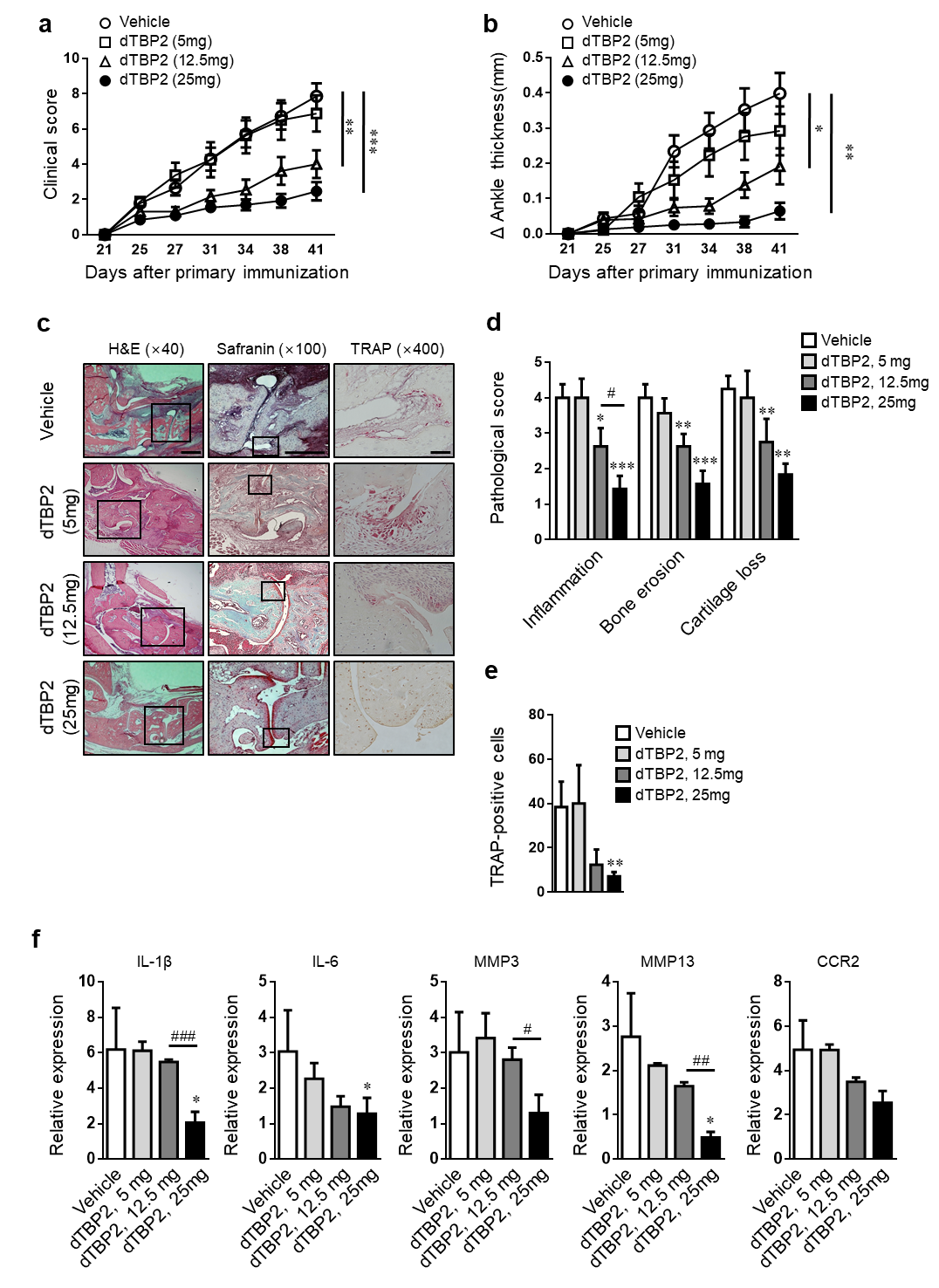
**

**Supplementary figure 2**

**
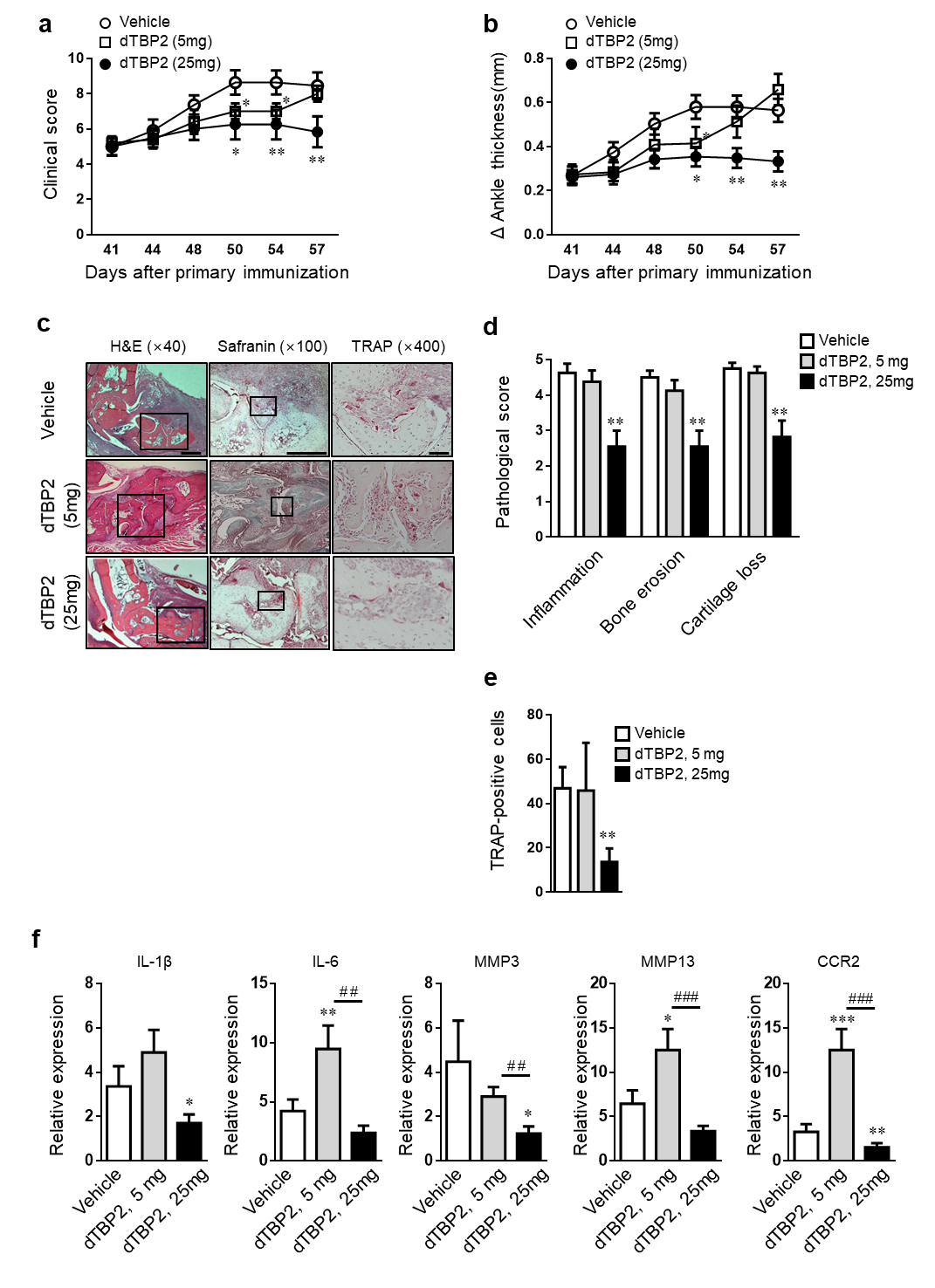
**

**2. Supplementary figure legend**

**Supplementary Figure 1. Prevention of inflammatory arthritis by dimerized HRF/TCTP-binding peptide (dTBP2).**

After generation of collagen-induced arthritis (CIA), the mice were treated with intraperitoneal injection of the vehicle or dTBP2( 5, 12.5 and 25mg.kg). **a, b** Mean clinical scores **(a)** and ankle thickness change **(b)** of CIA mice treated with the vehicle or dTBP2. **c** Ankle tissues, stained using H&E and safranin-O (bar = 500 μm), and immunolabeled for TRAP (bar = 50 μm). **d, e** Pathological scores for synovial inflammation, cartilage damage, and bone erosion **(d)**, and TRAP-positive cells in the ankle joints **(e)**. **f** Relative expression of inflammatory cytokines IL-1β, IL-6, MMP3, MMP13, and CCR2 in ankle joints, determined by qRT-PCR. Values are mean ± SEM (n = 10–12 mice for each group); * p < 0.05; ** p < 0.01; *** p < 0.001 versus vehicle-treated CIA mice; # p < 0.05; ## p < 0.01; ### p < 0.001 versus 12.5mg of dTBP2-treated mice.

**Supplementar Figure 2. Therapeutic effects of dimerized HRF/TCTP-binding peptide (dTBP2) on inflammatory arthritis.**

After generation of collagen-induced arthritis (CIA), the mice were treated with intraperitoneal injections of the vehicle or dTBP2 (5 and 25mg/kg). **a, b** Mean clinical score **(a)** and ankle thickness change **(b)** of CIA mice treated with the vehicle or dTBP2. **c** Ankle tissues, stained using H&E and safranin-O (bar = 500 μm), and immunolabeled for TRAP (bar = 50 μm). **d, e** Pathological scores for synovial inflammation, cartilage damage, and bone erosion, and TRAP-positive cells **(e)**. **f, g** Relative expression of inflammatory cytokines in the ankle joint. Values are mean ± SEM (n = 10–12 mice for each group); * p < 0.05; ** p < 0.01 versus vehicle-treated CIA mice; ## p < 0.01; ### p < 0.001 versus 5mg of dTBP2-treated mice.
